# Proteomic Signature of Frailty Pace Reflects Biological Aging and Predicts Disease and Mortality

**DOI:** 10.1093/geroni/igaf122.3833

**Published:** 2025-12-31

**Authors:** Jiachen Chen, Sandhya Iyer, Margaret Doyle, Joanne Murabito, Kathryn Lunetta

**Affiliations:** Boston University School of Public Health, Boston, Massachusetts, United States; Boston University School of Medicine, Boston, Massachusetts, United States; University of Vermont, Colchester, Vermont, United States; Boston University Chobanian & Avedisian School of Medicine and Boston Medical Center, Boston, Massachusetts, United States; Boston University School of Public Health, Boston, Massachusetts, United States

## Abstract

Frailty is a clinical manifestation of aging characterized by reduced physiological resilience and increased vulnerability to adverse outcomes. Plasma proteomics offers potential for tracking biological aging, yet few studies have used it to quantify frailty progression over time. We developed a proteomic signature representing the pace of frailty, derived from longitudinal trajectories of weight, grip strength, and gait speed in the Framingham Heart Study (FHS) Offspring cohort (N = 2,078, mean age 67 (SD = 9), 55% female). Using elastic net regression on 2,799 plasma proteins measured by the OLINK platform, we constructed a 112-protein signature spanning pathways related to muscle contraction, extracellular matrix interactions, homeostasis, immune responses and inflammation, metabolism, and neuronal system development. We validated the signature in two independent cohorts with plasma proteins measured by OLINK: the FHS Generation 3 (N = 3,130, mean age 46 (SD = 9), 53% female) and UK Biobank (N = 44,949, mean age 57 (SD = 8), 54% female). The signature was informative across adulthood and significantly associated (FDR ≤ 0.05) with the incidence of 19 major chronic diseases, including cardiometabolic, renal, pulmonary, hepatic, anemia, neurodegenerative, cerebrovascular, musculoskeletal, and oncologic conditions, as well as with all-cause mortality and age-related phenotypes such as telomere length, frailty index, blood pressure, lung function, reaction time and fluid intelligence score in UK Biobank, independent of age and other relevant covariates. Our findings demonstrate that the proteomic pace of frailty captures multiple biological processes underlying physiological decline and may serve as a prognostic biomarker of biological aging.

